# Pathogens and Passengers: Roles for Crustacean Zooplankton Viruses in the Global Ocean

**DOI:** 10.3390/microorganisms11041054

**Published:** 2023-04-18

**Authors:** Alastair J. Roberts, Curtis A. Suttle

**Affiliations:** 1 Department of Earth, Ocean and Atmospheric Sciences, University of British Columbia, Vancouver, BC V6T 1Z4, Canada; 2 Department of Microbiology and Immunology, University of British Columbia, Vancouver, BC V6T 1Z4, Canada; 3 Department of Botany, University of British Columbia, Vancouver, BC V6T 1Z4, Canada; 4 Institute for the Oceans and Fisheries, University of British Columbia, Vancouver, BC V6T 1Z4, Canada

**Keywords:** crustacean zooplankton, virus transmission, virus discovery, viral mortality, zooplankton morality, zooplankton disease, zooplankton virus, viral ecology

## Abstract

Viruses infect all living organisms, but the viruses of most marine animals are largely unknown. Crustacean zooplankton are a functional lynchpin in marine food webs, but very few have been interrogated for their associated viruses despite the profound potential effects of viral infection. Nonetheless, it is clear that the diversity of viruses in crustacean zooplankton is enormous, including members of all realms of RNA viruses, as well as single- and double-stranded DNA viruses, in many cases representing deep branches of viral evolution. As there is clear evidence that many of these viruses infect and replicate in zooplankton species, we posit that viral infection is likely responsible for a significant portion of unexplained non-consumptive mortality in this group. In turn, this infection affects food webs and alters biogeochemical cycling. In addition to the direct impacts of infection, zooplankton can vector economically devastating viruses of finfish and other crustaceans. The dissemination of these viruses is facilitated by the movement of zooplankton vertically between epi- and mesopelagic communities through seasonal and diel vertical migration (DVM) and across long distances in ship ballast water. The large potential impact of viruses on crustacean zooplankton emphasises the need to clearly establish the relationships between specific viruses and the zooplankton they infect and investigate disease and mortality for these host–virus pairs. Such data will enable investigations into a link between viral infection and seasonal dynamics of host populations. We are only beginning to uncover the diversity and function of viruses associated with crustacean zooplankton.

## 1. The Roles of Viruses and Planktonic Crustaceans in Marine Ecosystems

Viruses are by far the most abundant biological entities in the ocean, but their impacts on most marine metazoans are unknown. Across marine ecosystems, viruses exert their influence through infection, shaping microbial communities, driving evolutionary adaptation and altering the flow of energy through food webs in the process [1,2,3,4]. Viral lysis maintains the species diversity of phyto- and bacterioplankton by preventing the prolonged dominance by a taxon [5,6,7] and some viruses have been implicated in the termination of phytoplankton blooms [7,8,9]. This lysis moves organic matter from the classical food web into the microbial loop in a process known as the viral shunt [1,10], modulating the efficiency of carbon export into the deep ocean [11] and paradoxically increasing bacterial and primary production [12,13]. Many viruses need not kill their hosts in order to be transmitted or have reservoir hosts in which their effects are benign, while others even confer an adaptive benefit [14]. In multicellular hosts generally, fatal disease is likely to be the exception rather than the rule in viral infection, as many strategies for replication and proliferation do not necessitate severe disease. Despite new bioinformatic tools [15] and the recent explosion in virus discovery [16,17], many viruses of marine multicellular eukaryotes remain uncharacterised. Additionally, some viruses discovered in meta-omic or environmental reads lack an assigned host, since the inference of host–virus relationships from sequencing data can be difficult [18,19,20]. As a result, viral discovery has outpaced our understanding of which viruses infect whom and what their ecological roles might be. There is therefore a need to identify the viruses of key groups of marine organisms and uncover their impacts on host populations and the communities in which they are situated. One such group is crustacean zooplankton, whose functional importance far outweighs our knowledge of their viruses.

Crustacean zooplankton are among the most abundant animals on Earth and belong to the Pancrustacea, a broad evolutionary group of arthropods that is proposed to encompass both insects and crustaceans [21]. Planktonic members of the Pancrustacea range in size from cladocerans that are less than half a millimetre in length, to large copepods and small shrimp over a centimetre long. Barnacles and larger crustaceans such as decapods also have planktonic larval and juvenile stages with different functional roles than their adult forms [22].

Crustacean zooplankton also play key roles as trophic links in food webs and in altering the cycling of organic matter in marine ecosystems. They consume bacteria, phytoplankton and other zooplankton, facilitating the transfer of nutrients and energy to higher trophic levels, but in the process also release dissolved organic matter (DOM) through sloppy feeding and particulate organic matter (POM) through faecal pellets, carcasses and moults. Mesozooplankton are estimated to consume from 4–12 Gt C y^−1^ (8–24%) of global primary production directly, in addition to another 3.4–11.5 Gt C y^−1^ indirectly by grazing unicellular zooplankton, or 15–44% of global epipelagic primary production [22,23,24,25]. Much of the consumed primary production is respired or lost through non-consumptive mortality and excretion [23]; zooplankton vertical migration and faecal pellet generation constitute an estimated 25–70% of global biological carbon sequestration overall, and as much as 50–90% at lower latitudes [26,27]. Estimates of POM export from sinking zooplankton are not well constrained due to the lack of knowledge of the magnitude and causes of non-consumptive mortality; there is especially little known about disease. As the connector between primary production and larger animals, changes in the abundance of zooplankton and the composition of zooplankton communities caused by disease could have cascading effects through the food web [22,28]. Understanding the magnitude and effects of disease on zooplankton mortality and community composition is necessary both to improve the accuracy of ocean production models and to obtain a complete picture of marine food webs.

Few viral diseases of crustacean zooplankton are characterised, and the magnitude of zooplankton mortality due to viruses is unknown, as is the potential impact of zooplankton as vectors of viral disease. In many cases where mortality of zooplankton has been measured, the cause of death is unclear and mortality estimates are poorly constrained. Proposed causes of unexplained zooplankton mortality include environmental stress, starvation, toxin exposure and disease. Viral diseases in some larger crustaceans are well documented [29,30], and some of these viruses are also found in planktonic freshwater and marine crustaceans [31,32,33,34,35,36,37]; however, their impacts are understudied and largely a mystery in hosts that are not harvested for food. Additionally, even though zooplankton are known vectors of aquaculture and phytoplankton pathogens [36,37,38,39,40], the suite of pathogenic viruses harboured by abundant zooplankton is not established.

Viruses infect all living organisms, but the viruses infecting or transmitted by crustacean zooplankton and their effects on marine food webs are understudied. In this review, we summarise what is known about these pathogens and propose that widespread viral disease likely accounts for some of the non-consumptive mortality in crustacean zooplankton, with consequences for biogeochemical cycling. We also present examples of crustacean zooplankton as vectors of both fish and crustacean viruses and mechanisms of potential disease transmission. Finally, we propose approaches to (1) link viruses to zooplankton disease and mortality, (2) examine the roles of viruses in regulating zooplankton populations and (3) uncover the impacts of crustacean zooplankton as vectors of marine viruses.

## 2. The Pathogens: Viral Disease in Crustacean Zooplankton and Its Impacts

Despite being relatively understudied, the presence of diverse viruses in the crustacean zooplankton which have been examined suggests that infection plays an important role in zooplankton communities. Most of this viral diversity has been documented in copepods, with limited evidence of replication and disease. As crustacean zooplankton contain diverse viruses and many lethal viral diseases of other crustaceans have been described, viral infection likely contributes to the unexplained non-consumptive mortality observed in this group. This mortality potentially alters zooplankton community composition, reduces prey availability to larger animals and lowers grazing pressure on primary producers and microzooplankton. These factors would also impact biogeochemical cycling and nutrient regeneration, since zooplankton carcasses, faecal pellets and sloppy feeding are important sources of POM and DOM in the ocean.

### 2.1. The Viruses of Crustacean Zooplankton

There are few investigations into the symptomatology of zooplankton viral diseases and a definitive link to host mortality has not been established in marine species. Many viruses associate with and infect crustacean zooplankton, and some replicate and cause disease (Table 1); however, arthropods have sophisticated innate and specific immunity making it difficult to demonstrate the link between infection, viral replication and disease [41,42,43]. Viruses that cause disease include large double-stranded (ds) DNA iridoviruses that infect the decapod shrimp *Acetes erythraeus* as well as several *Daphnia* species and some copepods [44,45,46,47]. Small single-stranded (ss) DNA circoviruses have been associated with local population declines of the freshwater amphipod *Diporeia*, which was also found to carry viral haemorrhagic septicaemia virus (VHSV), a major pathogen of finfish [32,48]. Gammarid amphipods also carry diverse RNA viruses, including chu-, bunya-, partiti- and picorna-like viruses, among others [49]. Viral diseases of large decapods in the wild [29] and in aquaculture [50,51] have been reviewed extensively; yet, the impacts of these viruses on the planktonic larval stages are unknown. These viruses could contribute to the high larval mortality rates observed in planktonic crustaceans [52,53]. Isopod parasites of the shrimp *Latreutes fucorum* and the shore crab *Hemigrapsus oregonensis* also carry viruses [54,55], but it is unclear if these viruses are shared by, acquired from or transmitted to the crustacean hosts.

The viruses associated with copepods, their symptomology and prevalence in host populations have been recorded in some cases. For example, in Tampa Bay, Florida, the calanoid copepods *Acartia tonsa* and *Labidocera aestiva* are infected by the circoviruses Acartia tonsa copepod circo-like virus (AtCopCV) and Labidocera aestiva copepod circo-like virus (LaCopCV), respectively [56]. In different locations in Tampa Bay, LaCopCV was estimated to occur in 50 to 100 percent of host animals, with average viral loads ranging between 10^2^ and almost 10^6^ copies individual^−1^. By contrast, AtCopCV was not as commonly detected in *Acartia tonsa*, but did show seasonal peaks in prevalence, correlated with *A. tonsa* population increases and declines [56]. In 2013, Eaglesham and Hewson reported a similar pattern of detection of these two viruses over a larger range of sampling sites [57].

**Table 1:** Viruses associated with or infecting crustacean zooplankton.

Virus Name	Virus Order, Family	Associated Organism Order—Family, Genus or Species	Evidence of Replication	Disease Caused	References
Daphnia iridescent virus 1 (DIV-1)	*Pimascovirales*, *Iridoviridae*	Branchiopoda—*Daphnia* sp.	Yes	White fat cell disease	[47]
Unnamed circo-like viruses	*Cirlivirales*, *Circoviridae*	Amphipoda—*Diporeia* sp.	No	ND	[48,58]
Labidocera aestiva copepod circovirus (LaCopCV)	*Cirlivirales*, *Circoviridae*	Copepoda—*Labidocera aestiva*	Yes	ND	[56]
Acartia tonsa copepod circovirus (AtCopCV)	*Cirlivirales*, *Circoviridae*	Copepoda—*Acartia tonsa*	Yes	ND	[56]
Zooplankton invertebrate iridescent virus (ZoopIIV(HR))	*Pimascovirales*, *Iridoviridae*	Copepoda—*Gladioferens pectinatus*, *Boeckella triarticulata*, *Gippslandia estuarina*	Yes	Zooplankton iridoviral disease	[46]
White spot syndrome virus (WSSV)	Unclassified, *Nimaviridae*	Copepoda—*Acartidae*, *Oithonidae*, *Tachididae*, *Centropagidae*, *Corycaeidae*, *Temoridae*, *Calanidae*, *Paracalanidae*, *Pontellidae*, *Peltididae*, *Metrididae*, *Miraciidae*, *Cyclopidae*, *Ameiridae*, *Pseudodiaptomidae*, *Ergasilus manicatus* Amphipoda—*Caprellidae*, *Gammaridea* Cirripedia—undocumented family Branchiopoda—*Artemiidae* Mysida—*Mysidae* Euphausiacea—*Euphausiidae*	Variable	White spot disease	[33,35,38,39,59,60,61,62,63,64]
Viral haemorrhagic septicaemia virus (VHSV)	*Mononegavirales*, *Rhabdoviridae*	Amphipoda—*Diporeia* sp.	No	Viral haemorrhagic septicaemia	[32]
Unnamed mononega-like viruses	*Mononegavirales*, *Artoviridae*	Copepoda—*Caligus* spp., *Lepeophtheiris* spp., *Tracheliastes* sp., *Pseudodiaptomus* sp. Amphipoda—*Eulimnogammarus* sp., *Ommatogammarus* sp., *Hyatellopsis* sp.	No	ND	[49]
Unnamed mononega-like viruses	*Mononegavirales*, *Rhabdoviridae*	Copepoda—*Caligus* spp. Amphipoda—*Carinurus bicarinatus*, *Gammarus* spp.	No	ND	[49]
Unnamed chu-like viruses	*Jingchuvirales*, *Chuviridae*	Copepoda—*Cosmocalanus darwinii*, *Pleuromamma* sp., *Platychelipus littoralis* Amphipoda—*Hyatellopsis* sp., *Gammarus* sp.	No	ND	[49]
Unnamed ghabri-botybri-like viruses	*Ghabrivirales*, *Totiviridae*	Copepoda—*C. darwinii*, *Lepeophtheiris* spp., *Caligus* spp., *Tigriopus californicus*, *Labidocera madurae*, *Tracheliastes polycolpus* Amphipoda—*Gammarus* sp., *Eogammarus* sp., *Echinogammarus* sp.	No	ND	[49]
Unnamed tymo-like viruses	*Tymovirales*, *Tymoviridae*	Copepoda—*C. darwinii*	No	ND	[49]
Unnamed bunya-like viruses	*Bunyavirales*, unnamed families	Copepoda—*Lepeophtheirus* sp., *C. darwinii* Amphipoda—*Talitrus* sp., *Gammarus* sp., *Eogammarus* sp., *Hyatellopsis* sp.	No	ND	[49]
Unnamed orthomyxo-like viruses	*Orthomyxsovirales*, unnamed families	Copepoda—*T. californicus* Amphipoda—*Gammaroporeia* sp., *Platychelipus* sp., *Echinogammarus* sp., *Marinogammarus* sp.	No	ND	[49]
Unnamed qin-like viruses	*Muvirales*, *Qinviridae*	Copepoda—*Apocyclops royi* Amphipoda—*Gammarus* sp.	No	ND	[49]
Unnamed partiti-like viruses	*Durnavirales*, *Partitiviridae*	Copepoda—*C. darwinii*, *Caligus* spp., *T. californicus*, *Euchaeta* spp., *Eucalanus bungii* Amphipoda—*Eugammarus* sp., *Echinogammarus* sp.	No	ND	[49]
Unammed picobirna-like viruses	*Durnavirales*, *Partitiviridae*	Copepoda—*Eurytemora affinis* Amphipoda—*Talitrus saltator*	No	ND	[49]
Unnamed durna-like viruses	*Durnavirles*, *unnamed family*	Amphipoda—*Gammarus* sp.	No	ND	[49]
Unnamed martelli-like viruses	*Martellivirales*, *Endornaviridae*	Copepoda—*Caligus* sp.	No	ND	[49]
Unnamed picorna-like viruses	*Picornavirales*, unnamed family	Copepoda—*Caligus* spp., *Lepeophtheirus* sp., *E. bungii*, *Eucyclops serrulatus*, *Calanus finmarchicus* Amphipoda—*Gammarus* sp., *Echinogammarus* sp., *Ommatogammarus* sp., *Macropereiopus parvus*	No	ND	[49]
Taura syndrome virus	*Picornavirales*, *Dicistroviridae*	Copepoda—*E. manicatus*	Yes	Taura syndrome	[35]
Unnamed flavi-like viruses	*Amarillovirales*, *Flaviviridae*	Amphipoda—*Gammarus* sp.	No	ND	[49]
Unnamed noda-barna-like viruses	*Nodamuvirales*, *Nodaviridae*	Copepoda—*T. californicus*, *Pleuromamma abdominalis*, *E. affinis* Amphipoda—*Gammarus* spp.	No	ND	[49]
Unnamed noda-barna-like viruses	*Sobelivirales*, *Solemoviridae*	Copepoda—*Caligus* spp.	No	ND	[49]
Unnamed nido-like viruses	*Nidovirales*, unnamed family	Copepoda—*E. affinis*	No	ND	[49]
Unnamed birna-permutotetra-like viruses	*Durnavirales*, *Birnaviridae*	Copepoda—*E. affinis*	No	ND	[49]

ND = Not documented. For viruses for which evidence of replication differs among hosts, evidence of replication is marked as variable.

These data suggest that LaCopCV and AtCopCV may represent two different strategies of infection, in which LaCopCV is widespread, but mild or latent in its host, and does not generally cause severe disease or widespread mortality, while AtCopCV appears to be associated with periods of rapid growth or decay of host populations, possibly contributing to host mortality. Successful strategies for viruses exist on a continuum from mutualistic symbiont to latent disease to deadly pathogen [65,66,67], and crustacean viruses are expected to span this range as well.

Disease caused by viruses in the family *Iridoviridae* has also been documented across several different crustacean species in freshwater and marine environments. Iridoviruses are nucleocytoplasmic large DNA viruses (NCLDVs), which form paracrystalline arrays in infected tissue, giving afflicted individuals an iridescent or opalescent sheen. As such, iridovirus infection can be identified visually under the correct lighting. The *Iridoviridae* contains viruses that infect vertebrates and invertebrates, including fish (e.g., lymphocystis disease virus 1 and infectious spleen and kidney necrosis virus (ISKNV)), decapods (e.g., decapod iridescent virus 1 (DIV)) and cladocerans, in which Daphnia iridescent virus (DIV-1) causes white fat disease [44,45,47,68]. Suspected iridoviruses have also been imaged in the cells of ivory barnacle larvae [69]. Additionally, it is unclear whether a novel iridovirus recently identified in a deep-sea carnivorous sponge might infect its crustacean prey, which is plausible given the close relation of the virus to other invertebrate iridoviruses [70]. Iridovirus infection has been documented in copepods on the eastern coast of Australia, where symptomology was investigated extensively and prevalence of viral infection in estuarine copepods was recorded across time [46].

Several freshwater and estuarine copepods are persistently infected by iridoviruses in Victoria, Australia [46], suggesting that iridovirus infection may be widespread in copepods. The copepods displaying iridescence or opalescence were mainly *Gladioferens pectinatus*, but also *Boeckella triarticulata* and *Gippslandia estuarina*. Symptoms were reported to progress from weak opalescence or iridescence to strong blue phases, oedema and death. Paracrystalline arrays of virus particles were imaged by electron microscopy, indicating viral replication. Observationally, individuals also had reduced swimming and escape performance. Mortality was also observed, though isolation of the virus and reinfection of healthy, iridovirus-free individuals was not performed. High prevalence of iridovirus infection was correlated with increased water temperature and copepod density. Virus isolates from diseased copepods were either closely related to an invertebrate iridovirus of *Acetes* shrimp or to iridoviruses of vertebrates, specifically the lymphocysti- and ranaviruses which infect finfish. The latter case could be an example of past host switching, or of a virus with a wide host range. Host switching has been recorded when organisms are in prolonged close association [49,71,72]; switching of iridoviruses between fish and copepod parasites has been suggested [35], but no definitive evidence has been provided to date. Planktonic crustaceans are well situated to undergo host switching due to their ubiquity and ancient ecological associations with vertebrates and other organisms. Iridovirus infection of copepods may be a good model for exploring viral infection and dynamics in zooplankton, since advanced infection often makes hosts visible to the naked eye, expediting the screening of individuals for infection.

Diverse RNA viruses are also associated with planktonic and parasitic copepods [49] (Table 1). For example, sea lice, which have planktonic and parasitic stages, carry viral pathogens of fish; their role as vectors is discussed in the following section. Many RNA viruses have been discovered in copepods, but only a few species and samples have been examined and no symptomology or links to disease have been clearly established. Additionally, the prevalence of zooplankton-associated RNA viruses in host populations has not been reported. As copepods are globally abundant and integral to coastal and pelagic food webs, further attention should be given to RNA viral infection and disease in a broader range of copepod species.

### 2.2. Viral Disease Likely Accounts for a Portion of Non-Consumptive Mortality in Crustacean Zooplankton

Viral disease has largely been overlooked as a potential factor in zooplankton mortality. Estimates of zooplankton non-consumptive mortality are difficult to obtain, ranging widely with study method and conditions from less than 1 to over 80% [73,74,75]. Below, we review non-consumptive mortality in crustacean zooplankton and suggest that viral infection constitutes a portion of that mortality.

Myriad studies have estimated mortality rates of zooplankton, but difficulty in determining non-consumptive mortality has limited our ability to understand the regulation of zooplankton populations. The degree of non-predatory mortality and the fate of zooplankton carcasses is complex to measure; carcasses may be neutrally buoyant, be resuspended, be consumed after death or sink. Interspecies variation in tolerance to environmental stressors, changes over time in the causes of mortality, interannual variability in community composition, ocean conditions and disease pressure further complicate the estimation of mortality and its causes. Most studies have been conducted on copepods, and estimates vary widely with species, location and season [73,76]. For instance, some studies report high mortality of copepods in summer [77] and/or winter [74] in the Arctic [76] and at mid-latitudes [75,78], while another observed little difference between seasons at mid-latitudes in an abundant copepod species [79]. Suggested causes of zooplankton mortality include pH, oxygen and salinity stress, starvation, exposure to toxins, senescence and disease. Many of these causes also interact. For example, increased temperatures can lower tolerance to starvation [80] and hypoxia [81] due to increased metabolic demand. Higher temperatures and other environmental stressors are also usually associated with increased disease pressure [82,83].

Environmental stress caused by temperature, ocean acidification (OA), hypoxia and low salinity has been identified as a potential factor in copepod mortality and been given more attention in light of climate change. The impacts of these factors are species- and often stage-specific. For example, temperature stress and its effects on zooplankton mortality are expected to increase and result in future range shifts with ocean warming [73,84]. Similarly, although some zooplankton may not be substantially affected in the short term by present-day levels of OA [85,86], detrimental effects have been reported for pteropods [87], crab larvae [87] and krill [88]. These effects are made worse when pH stress is combined with low oxygen [89]. Ultimately, the complexity of conditions that plankton are exposed to in the wild makes it difficult to extrapolate laboratory-measured mortality and stress due to OA to natural communities [90]. Hypoxia is detrimental to zooplankton survival and reproduction as well [91,92,93], and is associated with changes in community structure [94]. Finally, salinity stress may affect mortality in fjords and estuaries with high freshwater input [95], though estuarine copepods are adapted to brackish water or can withstand short-term exposure to low salinities [96] despite paying an energetic cost to do so [97]. Thus, a variety of environmental stressors may contribute to non-consumptive mortality, whether directly or in synergy with disease or other stressors.

Massive die-offs of zooplankton in the winter have been attributed to either starvation or natural senescence post-reproduction, though disease is another potential cause. Insufficient lipid reserves has been proposed as an explanation for mass mortality of Arctic copepods, for example [74]. Copepods have vastly different starvation tolerances and metabolic needs [98]; insufficient lipid storage for overwintering may explain the high mortality and carcass flux observed in the winter when starvation stress is highest [99]. Senescence, on the other hand, is not likely to solely explain mortality, but may be a factor in deteriorating body condition that leads to other causes of mortality. Mortality of copepods increases with age and reproductive success, especially in males, suggesting a natural ageing process post-reproduction [100]. In some insects and copepods, immune function [101], disease resistance [102] and toxin resistance [103] are weaker in males and decline with age [104]. Senescence may make zooplankton vulnerable to mortality, but there is no evidence that it is the sole cause of mortality, and it does not account for the death of juveniles. It is plausible, however, that starvation leads to mass mortality in some species that overwinter, especially in the Arctic.

Finally, toxins produced by phytoplankton and death due to parasites kill zooplankton in some cases. Some diatoms can produce an aldehyde that inhibits hatching success and larval survival in co-existing planktonic copepods [105], and toxin production by harmful algal blooms (HABs) can deter or kill zooplankton; grazing can also increase toxin production by HABs [106]. Some zooplankton are resistant to algal toxins, while others avoid them by chemotaxis [107] or succumb to them [106]. For example, some dinoflagellates in the genus *Gyrodinium* produce a toxin that kills *Acartia* sp. [108]. Zooplankton also contain a wide range of endosymbionts and parasites [109], including dinoflagellates [109,110,111], ciliates [112,113], fungi [114], oomycetes and viruses [109,112,115,116]. Due to the conspicuous nature of ciliate and flagellate infection, some of these pathogens have been implicated in mortality. In one study, a parasitic *Paradinium* sp. killed up to a third of *Paracalanus* females [110], and ciliates have been documented causing mass mortality of euphausiids [112]. Pathogens causing severe disease might be hard to detect as diseased or dead zooplankton sink or are consumed.

Many different causes of non-consumptive mortality have been suggested to explain die-offs of crustacean zooplankton, but few clear links between a cause and death have been established. While environmental stressors, dietary shortfalls and cellular parasites might explain a portion of this mortality, viral infection is also likely important since viruses cause mortality in crustaceans and are found in zooplankton. Though definitive links to mortality have yet to be established, limited evidence of infection and observations of disease have been documented; thus, viruses likely account for some of the unexplained mortality in crustacean zooplankton. Documenting the significance of viral infection to non-consumptive mortality in zooplankton is a first step in understanding the role of viruses in regulating zooplankton populations and community structure, and ultimately its impact on trophic transfer efficiency and biogeochemical cycling.

### 2.3. Implications of Viral Disease for Food Webs and Biogeochemical Cycling

Viral infection is typically taxon-specific and density-dependent and therefore likely affects the composition of zooplankton communities by removing susceptible organisms. Conversely, resistance to infection would confer an advantage and may allow some taxa to persist or even colonise new areas. In turn, as functional roles differ among zooplankton species, changes in the taxonomic composition of the zooplankton community will potentially have cascading effects on the food web, pathways of trophic transfer and nutrient cycling. Though infection and disease will be influenced by environmental conditions, year-to-year variability and stochastic effects, viral infection that leads to zooplankton mortality will have predictable effects.

**Figure 1:**
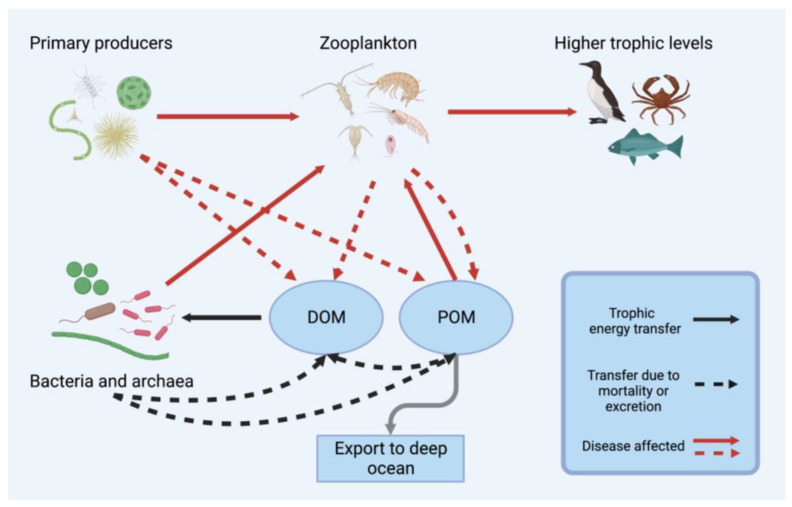
The effects of viral disease and transmission in crustacean zooplankton on pelagic food webs. Solid arrows represent trophic transfer, while dashed arrows represent the regeneration of organic material through mortality or excretion, e.g., cell lysis for primary producers or bacteria and carcasses or faecal pellets for zooplankton. Arrows coloured red represent relationships directly affected as a result of disease. Virus-caused mortality in crustacean zooplankton would result in reduced grazing pressure on primary producers and lower food availability to zooplankton predators. Reduced grazing would lower DOM generation from sloppy feeding but increase export of phytoplankton cells as POM. Mortality would increase POM flux from sinking carcasses but decrease the generation of faecal pellets which liberate DOM and contribute to POM generation.

Widespread viral disease in crustacean zooplankton would alter their availability to higher-level consumers as well as the grazing pressure they exert on phytoplankton and other prey, thus affecting the transfer of primary production to higher trophic levels. Total mesozooplankton production in epipelagic food webs is estimated at about 4.6 Gt C y^−1^, which supports the production of fish, seabirds and mammals [23]. Viral disease in zooplankton will affect the pathways of nutrient and energy flow in aquatic food webs (Figure 1). Infection can make hosts more vulnerable to predation if individuals become more conspicuous or have reduced predator escape ability, as has been inferred for infection by chytrids [117] and iridoviruses [46]. Increased predation could either reduce the spread of infection by removing infected individuals from the population or increase infection by enhancing the spread of free virus particles or infected tissue, as occurs in some animal systems [118]. As the nutritional value of zooplankton varies depending on their lipid content and composition [28,119], changes in the zooplankton community could affect the amount and quality of nutrients available to consumers [22,28,120]. Hence, disease outbreaks in zooplankton would affect higher trophic levels. Alterations in the abundance of particular zooplankton due to virus-caused mortality would also affect the primary producers, bacteria and zooplankton on which they prey [121]. Zooplankton grazing can prevent domination by specific phytoplankton and help maintain a diverse community of primary producers and microzooplankton [22,25]. In the same sense, viruses may also prevent dominance by specific taxa of zooplankton by suppressing abundant taxa, and differential susceptibility to abundant viruses in a particular region may confer an advantage to resistant taxa in dominating the community or colonising new regions. Changes in zooplankton community composition due to viral infection are likely to have cascading effects on food webs.

Zooplankton mortality influences the generation of different types of particulate organic carbon (POC) by zooplankton and the fate of that carbon. This includes increased POC generated by sinking carcasses and decreased POC generated by excretion of faecal pellets (Figure 1). Estimates of zooplankton mortality and the contribution of zooplankton carcasses to sinking POC range widely based on location, population bloom status, time of year and method of estimation; however, zooplankton mortality contributes significantly to carbon export [23,74,99,122,123,124]. For instance, estimates of copepod turnover and associated contribution to POC flux vary widely from 0–0.5 day^−1^ and 0–80%, respectively [74,99,122,123]. Carcasses produced due to viral disease could also spur microbial production [25]. High zooplankton densities are also likely to increase disease transmission and potentially disease severity [125,126,127]. While viral mortality might increase POC flux from carcasses, reduced populations of zooplankton would result in lower export of microzooplankton, bacteria and primary producer biomass through excretion as faecal pellets [23], a significant component of the biological carbon pump [26]. DOM generation from sloppy feeding and faecal pellets would also be reduced if infection led to less grazing, potentially lowering primary and bacterial production due to decreased nutrient regeneration.

The impact of zooplankton viruses on food webs and marine communities also includes the viruses that they carry that do not cause severe disease. Some crustacean viruses and pathogens are lethal, while others are sublethal and chronic or are resisted and eventually overcome [29,128]. Many viruses cause latent or mild infections which only cause severe disease under specific conditions, such as high host density or stress. Such viruses may be relatively benign in one host, but cause severe disease in another, as discussed below.

## 3. The Passengers: Viruses Spread by Crustacean Zooplankton

**Figure 2:**
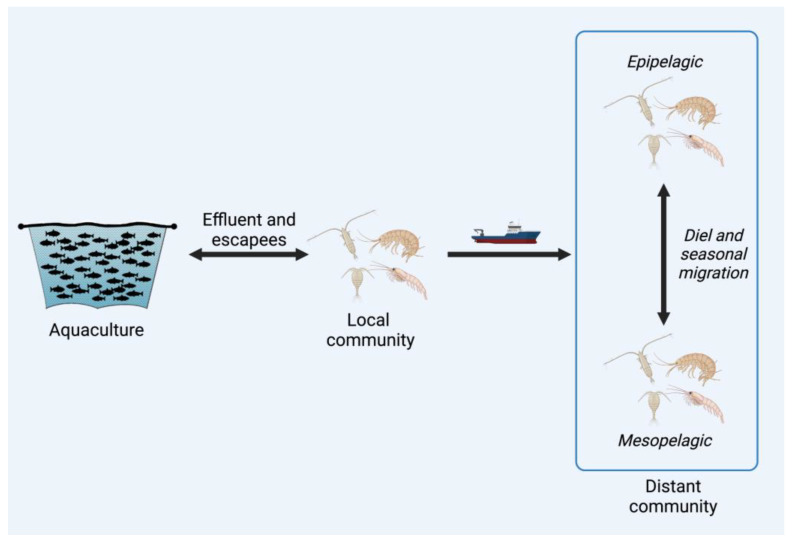
Crustacean zooplankton can vector viruses horizontally and vertically in the ocean. Viruses are transmitted among wild crustaceans or move between aquaculture pens and the local crustacean community through escapees, effluent or vectors. Viruses harboured in the wild are also transported into open net pens by zooplankton, where host density and environmental conditions are conducive to disease development. Diel or seasonal vertical migration of zooplankton transports organic matter between deep water and the mixed layer, with the potential to transmit pathogens including viruses. Long-distance transport of viruses carried by zooplankton in ship ballast should also examined as a mechanism of viral spread.

In addition to their own viruses, crustacean zooplankton carry and transmit viruses of other organisms. Examples include crustacean zooplankton spreading viruses between phytoplankton [129,130], in aquaculture [36,38,39,40,69], between wild fish [34,37,131] and to their predators [132]. Of all the marine invertebrate viruses described, those with direct economic impacts on aquaculture have been studied most extensively regarding their disease impacts and modes of transmission [30,50,51,133]. Pathogens in industrial aquaculture are a persistent and expensive problem, and viruses are responsible for yield losses, especially in shrimp farming [30,134,135]. Pathogens that proliferate in cultured fish and crustaceans can be reintroduced to wild communities through effluent or escaped organisms [136,137] with detrimental effects on the ecosystem [136,137,138,139,140,141]. Zooplankton can act as vectors between aquaculture and wild communities, as well as within or between wild communities, potentially facilitated by shipping (Figure 2). Although this process remains understudied overall, the best-known case of viral transmission and disease in crustaceans is that of white spot syndrome virus (WSSV), a double-stranded DNA virus of the *Nimaviridae* within the unassigned class *Naldaviricetes*.

White spot syndrome virus (WSSV) devastates crustacean aquaculture [30,142,143] and infects wild crustaceans, especially in proximity to aquaculture operations, where over 50% of wild organisms test positive for WSSV in some cases [64]. Higher water temperatures can trigger or exacerbate disease development of WSSV [144] and virus particles are remarkably stable in seawater [145], making it of particular concern for aquaculture operations as the ocean warms. The virus is widespread in copepods and other zooplankton in proximity to farms, which are established reservoir hosts for WSSV [39,63]. Many crustaceans can transmit WSSV to aquaculture from the local environment, or facilitate its spread within aquaculture, including barnacles [40], copepods [38] and mysid shrimp [62], amongst other crustaceans [29,31,50,60,146]. The virus is also able to ‘piggyback’ on phytoplankton, resulting in subsequent transmission to grazers [62]. Virulence and a wide host range make WSSV a devastating and persistent disease that is exacerbated by its crustacean vectors and reservoir species. There is a constant exchange of virus between aquaculture and local communities, facilitated in part by planktonic crustaceans [29,64,134]. While WSSV is the best documented, it is not the only economically important pathogen with a zooplankton reservoir.

Other crustacean and vertebrate viruses are vectored by zooplankton such as amphipods, but also sea lice, which have planktonic and parasitic stages. For example, the amphipod *Diporeia* carries viral haemorrhagic septicaemia virus (VHSV) in the Great Lakes [32,48,58]. Viral haemorrhagic septicaemia affects both marine and freshwater fish. It has been implicated in large fish kills in the Great Lakes [147] as well as in the mortality of herring and salmon in British Columbia, where it is thought to have been passed between wild and farmed fish [148]. Many fish and shrimp viruses are carried by sea lice or replicate within them, including taura syndrome virus (TSV) in *Ergasilus manicatus* [35], infectious haematopoietic necrosis virus (IHNV) in *Lepeophtheirus salmonis* [37] and infectious salmon anaemia virus (ISAv) in *Caligus rogercresseyi* [149]. Sea lice parasitise fish tissue, where blood feeding makes them ideal mechanical vectors. Crustacean zooplankton are globally distributed, some have been identified as vectors or reservoirs of devastating diseases of finfish and other crustaceans in aquaculture and the wild. As a result, the complement of viruses carried by these organisms should be identified and the mechanisms of their spread require further study.

Zooplankton, which are prolific vectors, are spread globally in ship ballast water. Ballast water is a source of non-indigenous zooplankton (NIZ) and viruses introduced to freshwater and marine environments [150,151,152,153,154,155], both of which can disrupt ecosystems if they establish successfully [150,156,157]. While there are regulations on ballast water exchange prior to port entry for international arrivals, some NIZ are still introduced, and domestic vessels can spread NIZ from previously invaded areas to uninvaded ones [158,159,160]. Further, though non-indigenous zooplankton are monitored for their potential ecological effects, their viruses and the viruses of zooplankton generally are mostly uncharacterised and not given adequate attention in risk assessments. Since zooplankton carry pathogenic viruses of other organisms and can be transported across oceans in ship ballast, their potential role in long-distance disease transmission should be examined.

In addition to long-distance horizontal transport, many zooplankton perform daily and seasonal vertical migrations through the water column, which may spread pathogens between communities at different depths. Up to 50% of zooplankton above 500 m perform diel vertical migration (DVM), rising to the surface to feed at night [161]. The diel and seasonal vertical movements of zooplankton transfer organic material between layers with high energetic barriers to mixing, connecting epi- and mesopelagic plankton communities and sequestering carbon [162,163,164]. Zooplankton are infected by and carry viruses, and infected individuals could release virus particles when they are migrating, consumed or dying. Copepod carcasses can be suspended and drift in the epipelagic layer with long residence times [79], sometimes reaching neutral buoyancy and remaining connected to the food web. These carcasses are food for scavenging organisms and are a potential source of pathogens if their contents are released [165]. For instance, drifting *Neocalanus cristatus* carcasses can make up 30–40% of the adult diet of *Euphausia pacifica* in the Sea of Japan [166]. The consumption and subsequent excretion of dead *N. cristatus* by *E. pacifica* redistributes the organic matter contained within carcasses; therefore, suspended zooplankton carcasses infected with viruses or other pathogens represent a potential inoculum that could initiate or prolong disease outbreaks. Zooplankton consumption of infected individuals or carcasses and the subsequent release of viral particles at varying depths during migration could spread viruses vertically in the water column. Faecal pellets may also act as vectors given that some viruses remain infectious after passing through the zooplankton digestive system [130]. Overall, vertical migration by zooplankton connects different water masses and ultimately epi- and mesopelagic communities. This regular movement of organic material may be a mechanism of pathogen dispersal for zooplankton as well as photo- and heterotrophic microbes that has yet to be investigated.

Crustacean zooplankton can be reservoirs and vectors of economically damaging viruses. Identifying the viruses associated with and infecting crustacean zooplankton is necessary to monitor the impacts of these viruses on aquaculture and wild communities and understand how they spread on local and global scales. The full complement of viruses carried by these organisms warrants further attention in order to better understand zooplankton life cycles and ecology, the mechanisms of disease transmission in the ocean and the disease transmission risks associated with shipping.

## 4. Lifting the Veil on the Identity and Impacts of Zooplankton Viruses

Our understanding of viral diversity and evolution in the ocean has massively increased over the past decade, leading to the establishment of entirely new phyla [17] and major taxonomic adjustments by the International Committee on the Taxonomy of Viruses (ICTV) [167]. This has been spurred by metagenomic sequencing of environmental samples and in select hosts of interest, leaving the viruses of large swaths of ocean organisms neglected, including crustacean zooplankton, a functional lynchpin in ocean production. While there are some documented examples of viruses infecting zooplankton, much of the diversity of viruses in this group remains unexplored. Characterising these viruses and their impacts will allow viral infection of zooplankton to be integrated into our understanding of biogeochemical cycling, energy flow through food webs and disease transmission within and external to crustacean zooplankton. Moving forward, we propose that the following approaches are warranted to investigate the diversity and impacts of crustacean zooplankton viruses:

*I*. *Virus discovery in crustacean zooplankton with a focus on the establishment of host–virus relationships as well as a link between infection, disease and mortality*.

Virus discovery efforts may include primer-based interrogations of plankton samples for specific viral groups, or meta-omic approaches to capture a larger snapshot of viral diversity. There is no known host association for most viruses identified in environmental samples or those found in meta-omic reads from tissues. Giving evidence of viral replication in a host is challenging, but it can be accomplished using strand ratios from transcriptome reads, host–virus co-divergence analysis [49], through proximity ligation methods [20] or by examining immune markers of active infection. Correlational evidence for mortality can be obtained by linking viral prevalence or concentrations in host populations with death, and molecular probes can be used to interrogate dead zooplankton for evidence of viral infection. Ideally, disease transmission can be shown by infecting healthy animals with viruses from infected individuals, and then recovering the same virus after disease induction. This process could be challenging due to the difficulty in culturing many zooplankton, which have complex lifecycles, as well as the fact that crustaceans can be infected by and carry multiple viruses which may not cause severe or detectable disease unless presented with the right conditions within the host. Establishing host–virus systems in culture will allow thorough investigations into symptomatology and virulence as well as host immune response and resistance.

*II*. *An assessment of viral prevalence, distribution and seasonal dynamics in crustacean host populations*.

The host ranges and prevalence of viruses in crustacean zooplankton are still unclear. Once viral genomes and host–virus pairs are better known, the occurrence and impacts of viruses on zooplankton populations can be investigated. Comparisons between the presence of viruses and mortality could shed light on viral infection strategies in zooplankton. Molecular probes could be used to assess virus occurrence in host populations across space and time. Spatial data would allow for investigations into distribution and transmission across long distances. Temporal data will be essential in determining if viruses play a role in zooplankton seasonal mortality and identifying which viruses are persistent or ephemeral. The interrogation of temporal samples for viruses represents a possible role for virologists in working with well-established zooplankton time series.

*III*. *A comprehensive assessment of viral ‘passengers’, mechanisms of transmission and associated risks*.

As described above, crustacean zooplankton can be vectors of lethal pathogens of fish, crustaceans and phytoplankton. Further attention should be paid to the potential for zooplankton to spread viruses vertically through the water column and horizontally across large distances. The roles of DVM, seasonal migration and global shipping in spreading crustacean viruses warrant investigation. The first step in assessing the role of vertical movement by zooplankton in virus dissemination is to identify the viruses carried by zooplankton and determine when and how they are shed. Once candidate viruses are identified, assays for zooplankton-associated viruses in the water column can also be performed. If pathogens of concern are identified in zooplankton, monitoring programs for ships should be expanded to include these viruses.

## 5. Conclusions

Crustacean zooplankton are taxonomically diverse and abundant, yet their viruses remain undiscovered and the impacts of those viruses are largely unknown. Viruses associated with zooplankton include representatives from deep branches of viral evolution and employ a range of infection strategies including causing severe disease. Viruses are expected to play a substantial role in zooplankton mortality, with disease influenced by environmental factors, although evidence remains sparse. Zooplankton growth and mortality models are poorly constrained as the fate of zooplankton biomass in the ocean is not well-established, a missing component in our understanding of how populations are regulated. This limits our ability to accurately assess the role of zooplankton in biogeochemical cycling and how viruses impact the nutritional landscape available to fish and larger animals. Zooplankton are also prolific viral vectors whose viruses must be identified before their impacts on local and global disease transmission can be assessed. The viruses associated with zooplankton and their impacts represent an unknown frontier of diversity and ecological interactions in the ocean that has not been given adequate attention.
